# Bisindolylmaleimides New Ligands of CaM Protein

**DOI:** 10.3390/molecules27217161

**Published:** 2022-10-23

**Authors:** Alejandro Sosa-Peinado, Karina Fructuoso-García, L. X. Vásquez-Bochm, Martin González-Andrade

**Affiliations:** 1 Laboratorio de Biosensores y Modelaje Molecular, Departamento de Bioquímica, Facultad de Medicina, Universidad Nacional Autónoma de México, Ciudad de México 04510, Mexico; 2 Departamento de Farmacia, Facultad de Química, Universidad Nacional Autónoma de México, Ciudad de México 04510, Mexico

**Keywords:** biosensors, calmodulin, bisindolylmaleimides, anti-CaM drugs, docking, molecular dynamic, chemoinformatic

## Abstract

In the present study, we reported the interactions at the molecular level of a series of compounds called Bisindolylmaleimide, as potential inhibitors of the calmodulin protein. Bisindolylmaleimide compounds are drug prototypes derived from *Staurosporine*, an alkaloid with activity for cancer treatment. Bisindolylmaleimide compounds II, IV, VII, X, and XI, are proposed and reported as possible inhibitors of calmodulin protein for the first time. For the above, a biotechnological device was used (fluorescent biosensor *h*CaM M124C-*mBBr*) to directly determine binding parameters experimentally (*K*_d_ and stoichiometry) of these compounds, and molecular modeling tools (Docking, Molecular Dynamics, and Chemoinformatic Analysis) to carry out the theoretical studies and complement the experimental data. The results indicate that this compound binds to calmodulin with a *K*_d_ between 193–248 nM, an order of magnitude lower than most classic inhibitors. On the other hand, the theoretical studies support the experimental results, obtaining an acceptable correlation between the ΔG_Experimental_ and ΔG_Theoretical_ (r^2^ = 0.703) and providing us with complementary molecular details of the interaction between the calmodulin protein and the Bisindolylmaleimide series. Chemoinformatic analyzes bring certainty to Bisindolylmaleimide compounds to address clinical steps in drug development. Thus, these results make these compounds attractive to be considered as possible prototypes of new calmodulin protein inhibitors.

## 1. Introduction

**Figure 1:**
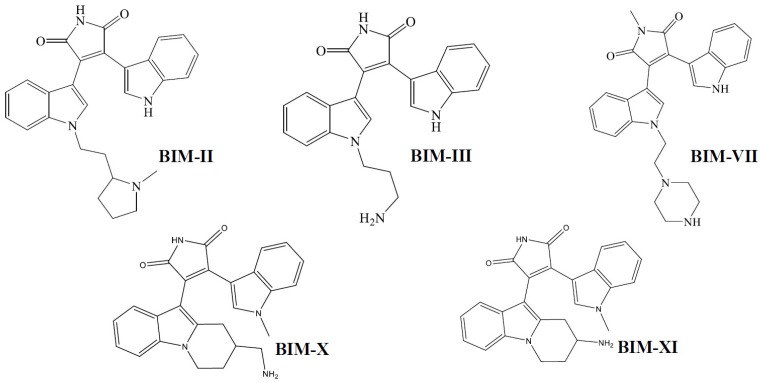
Chemical Structures of BIMs.

Bisindolylmaleimides (BIMs) are organics compounds derivates from indolocarbazoles, *staurosporine* formed by a maleimide group, and two indole groups bound to it [1]. A series of compounds have been synthesized from BIMs with different substituents in one of the indoles (Figure 1); this series includes BIM from I to XI; biological activity has been reported for these compounds. BIM I has been reported as an inhibitor of protein kinase C (PKC) [2] and glycogen synthase kinase 3 (GSK3) [3], as well as a competitive antagonist for the 5-HT3 receptor [4]. BIM II is a general inhibitor of all PKC [5]. BIMs I, II, III, IV, and V interact and inhibit ABCG2 (a transporter with potential importance in cancer drug resistance) [6]. BIM IV, in addition to inhibiting the PKC, also inhibits the cAMP-dependent protein kinase (PKA) [5]. BIM I, BIM II, BIM III, BIM VI, BIM VII, and BIM-VIII inhibit solute carrier organic cation transporter (OCT) 1, involved in the uptake of marketed drugs in the liver [7]. BIM IX is a potent inhibitor of GSK-3 [3]. BIM X is also considered an inhibitor of protein kinases (PKs) [8]. BIM XI inhibits PKC and prevents T-cell activation and proliferation [9,10]. In most of these trials, they are carried out by competitive or coupled studies, where the BIMs can interact in more than one molecular target to show the reported effects.

**Figure 2:**
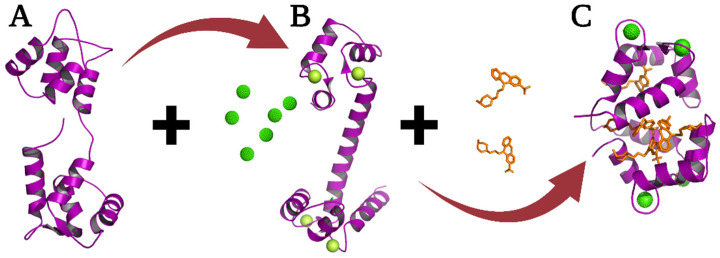
Structure three-dimensional models of the CaM in its three main conformations. Apo-CaM (**A**) was resolved by NMR (1CFD.pdb), Holo-CaM (**B**) with its four occupied binding sites shown in green spheres (1CLL.pdb), and the Ca^+2^-CaM-TFP complex (**C**) corresponding to the closed form of the protein (1LIN.pdb).

A molecular target drug that interacts with many molecules and hence regulates many metabolic pathways is the protein calmodulin (CaM). In recent years, the CaM has been the subject of various studies, including computational, thermodynamic, structural, evolutionary, and pharmacological [5,6,7,8,9,10,11]; this protein is one of the most abundant, ubiquitous, and conserved, and more than 60% of which are conserved among eukaryotes and 100% among vertebrates [12]. The sequence of CaM comprises 148 amino acids (16.7 KDa) formed by two domains containing each domain two Ca^2+^-binding loops known as EF-hand. CaM has no enzymatic activity but plays an essential role in calcium signaling pathways. CaM interacts with many proteins to activate or regulate the concentration of calcium intracellular [11,12]. CaM adopts three important conformations: Apo-CaM in the absence of calcium, Holo-CaM after binding Ca^+2^ ions where it exposes hydrophobic patches which are essential for the interaction and modulation of other proteins, and finally, the closed conformation where the protein interacts with its inhibitors (drugs or peptides) and in this conformation it is unable to perform its function (Figure 2) [13,14]; this protein is a molecular target of compounds with pharmacological activity, such as anti-cancers, antipsychotics, antidepressants, muscle relaxants, and local anesthetics. Moreover, it is involved in physiological processes such as muscle contraction, fertilization, cell proliferation, vesicular fusion, apoptosis, and others [15,16,17,18,19].

Studies of the physicochemical and structural properties of various CaM inhibitors have shown ionic and hydrophobic interactions between the ligands and CaM. The structural relationships between these ionic and hydrophobic regions and other factors such as the structure of the ligands (mainly resonant rings, large and hydrophobic structures, and halogens present), ionic forces, and electrostatic interactions, so far identified, are important and necessary factors for binding. Of molecules to CaM, not only is an isolated characteristic necessary for the correct interaction, some characteristics may even dominate more than others, but that depends on the nature of the compound itself [20]. Many known compounds that inhibit CaM have structural similarities, suggesting that the geometric structure of a bioactive molecule is essential in determining its interaction with CaM. In many cases, slight modifications in chemical structure can significantly alter a compound’s ability to bind to CaM and inhibit its activity. A direct detection tool developed by our group is the calmodulin biosensor (*h*CaM-M124C-*mBBr*), which we have used to detect the binding of various ligands [21,22,23,24,25,26,27]. On the other hand, theoretical and computational studies, such as cheminformatics, docking, and molecular dynamics simulations (MD), are considered tools of great value to complement the experimental data. Therefore, this work uses experimental techniques and computational tools to propose new CaM inhibitors as possible prototypes of anti-CaM drugs.

## 2. Results and Discussion

### 2.1. Determination of the K_ds_ of the BIM Compounds Using the Fluorescent Biosensor hCaM-M124C-mBBr

**Figure 3:**
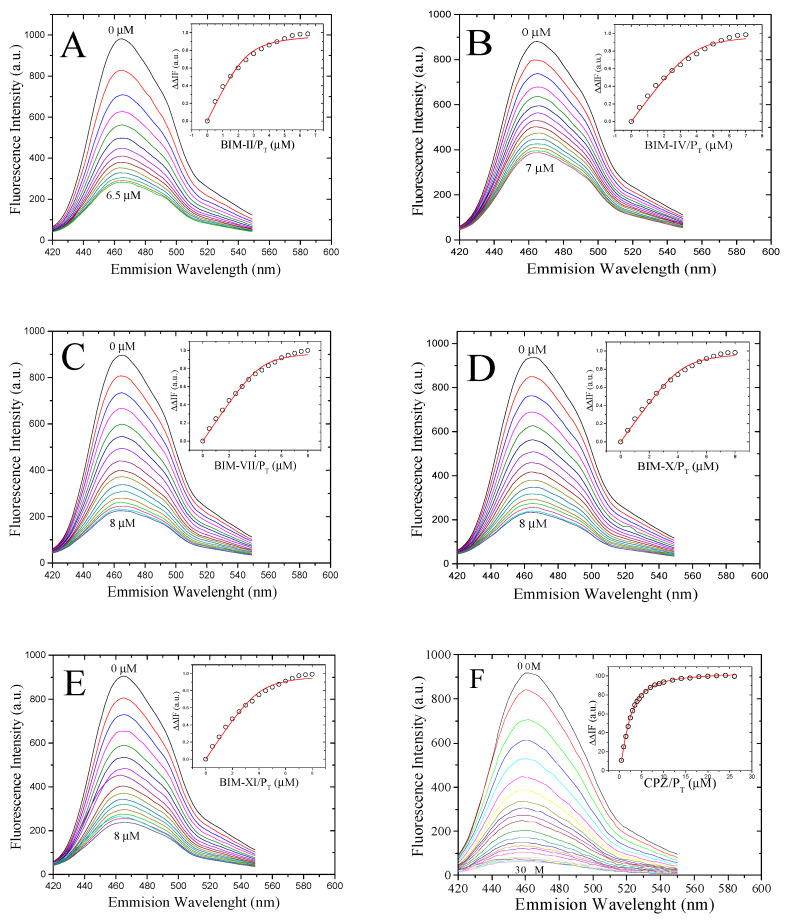
Fluorescence spectra and titration curves of Ca^+2^-*h*CaM M124C-*mBBr* with BIM-II (**A**), BIM-IV (**B**), BIM-VII (**C**), BIM-X (**D**), BIM-XI (**E**), and CPZ (**F**). Buffer was 10 mM of potassium acetate pH 5.1 at 37 °C. The absolute changes of maximal fluorescence emission were corrected for light scattering effects and plotted against the ligands to total protein ratio (insets). The continuous line in the insets comes from data fitting to the binding model (Equation (1) in the experimental section) to obtain the *K*_d_.

Figure 3 shows the BIMs and Chlorpromazine (CPZ) spectra and fluorescence titrations with the *h*CaM-M124C-*mBBr* biosensor; this device can measure the direct interaction of CaM ligands with great sensitivity and obtain binding parameters (*K*_d_ and stoichiometry). All compounds exhibit a quenching of fluorescence intensity upon interaction with CaM; this may be the beginning of the conformational change that CaM undergoes when interacting with these ligands. The estimated K_ds_ are in the nM order, with the following affinity order: BIM-VII > BIM-XI > BIM-IV > BIM-X > BIM-II > CPZ (Table 1); which makes this series of compounds desirable to be considered as possible anti-CaM drugs. Many of the inhibitors reported so far are in the micromolar range, such as KAR-2 (5 mM) [28], Imipramine (14 mM) [29], Serotonin (0.71 mM), Chlorpromazine (0.97 mM) [26], Trifluoperazine (1 mM), W7 (7 mM) [30] and Lubeluzole (2.9 mM) [31]; only some peptides are in the nanomolar range [32]. The stoichiometry of the compounds is from the ratio of 1:2 to 1:4, which is mainly attributed to the size of each compound.

### 2.2. Docking Studies of the BIMs Series with the CaM Protein

**Figure 4:**
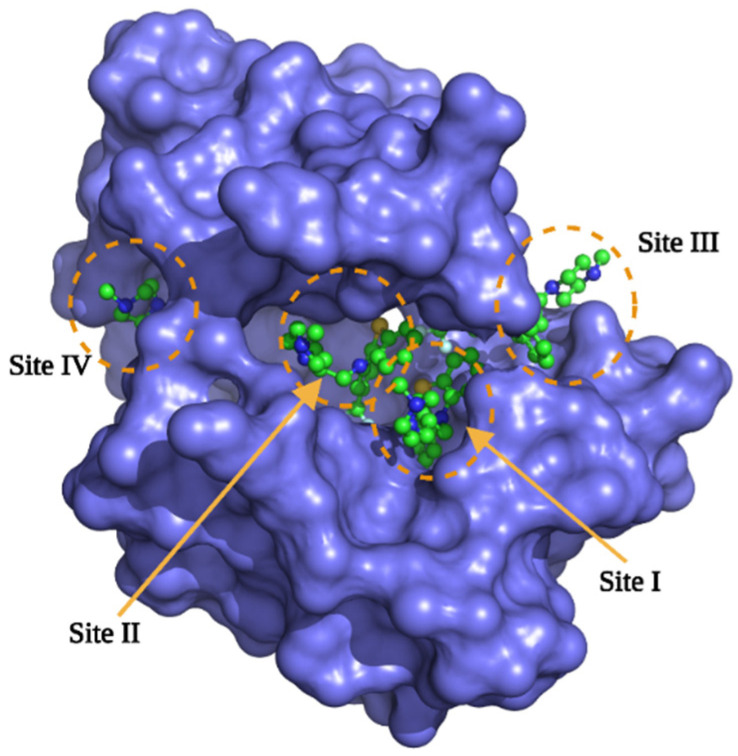
Structural model of the CaM-TFP 1:4 complex, from the 1LIN.pdb code. Sites were designated according to the degree of affinity based on Dockig’s studies.

**Figure 5:**
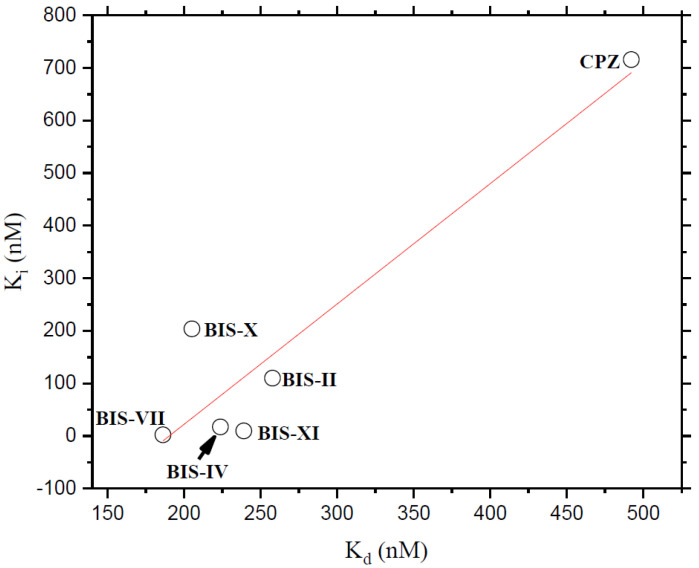
Relationship between experimental *K*_d_ and theoretical *K*_i_ of the BIM ligands.

Based on the experimental results, we carried out docking studies of the BIMs compounds against the CaM. It has been reported that CaM can bind to more than one ligand depending on its size; for example, the co-crystallized CaM-TFP complex is available in a 1:2 (1A29.pdb) or 1:4 (1LIN. pdb). Figure 4 shows the four trifluoperazine (TFP) binding sites on CaM. We calculate the theoretical *K*_i_ of each TFP in the four positions, and based on the affinity; we designate the sites; thus, site I is the one with the best affinity, and site IV has the lowest affinity for TFP. Results are presented in Table 1; depending on the stoichiometry of the BIMs and CPZ, theoretical *K*_i_ was calculated for sites I, II, III, or IV. Four sites behave according to the TFP calculation. Site I shows the best affinity in all cases, and site IV presents the lowest affinity. A graph was constructed to establish a relationship between the calculations obtained experimentally and theoretically (Figure 5), using the experimental *K*_d_ and the theoretical *K*_i_ of site I, showing a linear relationship between the data with an r^2^ of 0.85; this correlation is good since, based on it, we can carry out a structure-function analysis of the BIMs series and address the interactions between the Ca^+2^-CaM-BIM complexes in detail. Experimental and docking data indicate that the affinity of the BIMs compounds is in the nM range.

**Figure 6:**
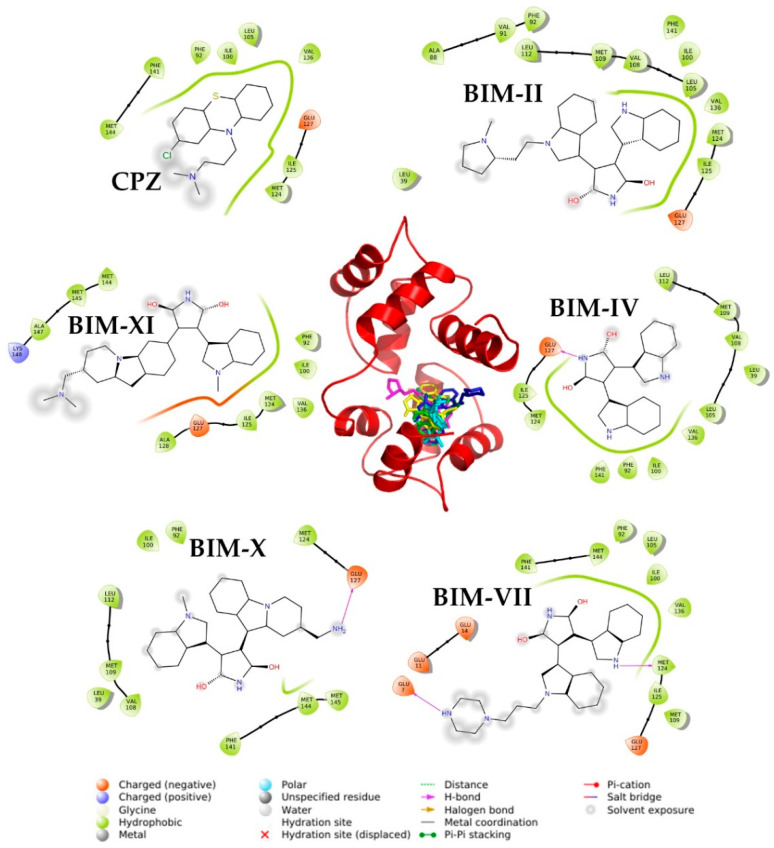
Structural model of docking results with BIMs compounds in site I of the CaM. The interactions with the residues at 4 Å are shown in the periphery. The image was made with PyMOL and Maestro.

Details at the molecular level of different Ca^+2^-CaM-BIMs complexes are shown in Figure 6 and Table 2; all the ligands bind in the same region, made up of primarily hydrophobic residues (Table 2). Theoretical *K*_i_, calculated by AutoDock4 presented in Table 3, where the BIM-VII compound showed the highest affinity (*K*_d_ = 2.14 nM) and the most significant number of contacts with negatively charged residues (Four Glu for this case); while for the rest of the ligands they have only 1. Additionally, this ligand forms two hydrogen bonds with residues Glu7 and Met124. BIM-VII is the ligand that presented the lowest *K*_d_ in the experimental binding studies (186.2 nM), which is in harmony with these theoretical results; this same tendency can be observed with the positive control (CPZ), where the *K*_d_ and *K*_i_ are the highest at 492 and 715 nM, respectively.

### 2.3. Molecular Dynamics Simulation Studies

**Figure 7:**
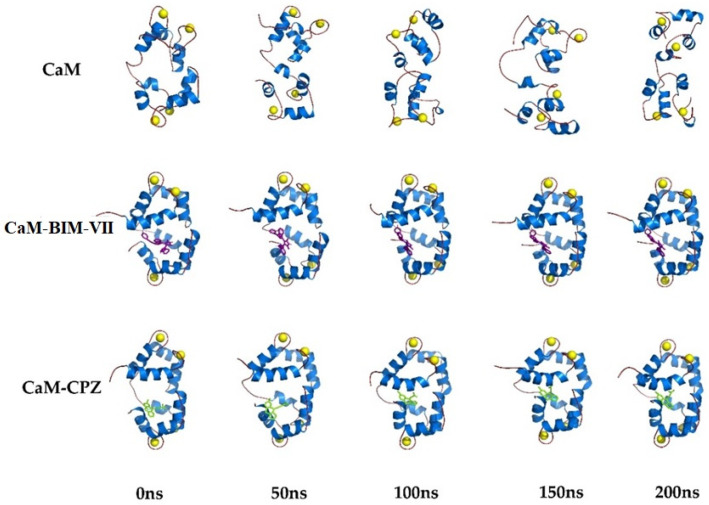
Structural models of molecular dynamics simulation of Ca^2+^-CaM and Ca^2+^-CaM-BIS-VII and, Ca^2+^-CaM-CPZ complexes (200 ns).

Molecular Dynamics (MD) studies of the Ca^2+^-CaM-BIM complexes were carried out to obtain dynamic-structural and energetic information about this series of compounds. MD was performed up to 200 ns, a reasonable time to evaluate the desired energetic and structural parameters. Figure 7 shows the structural models generated from the MD trajectories for Ca^2+^-CaM, Ca^2+^-CaM-BIM-VII, and Ca^2+^-CaM-CPZ complexes. We can observe that the protein is in the closed state without ligands and, after 50 ns tends to be open. In comparison, the complexes remain closed for at least 200 ns; this behavior can be attributed to the ligands providing stability to the closed CaM conformation. At the same time, the free protein can transit between the closed and open states in a dynamic equilibrium.

**Figure 8:**
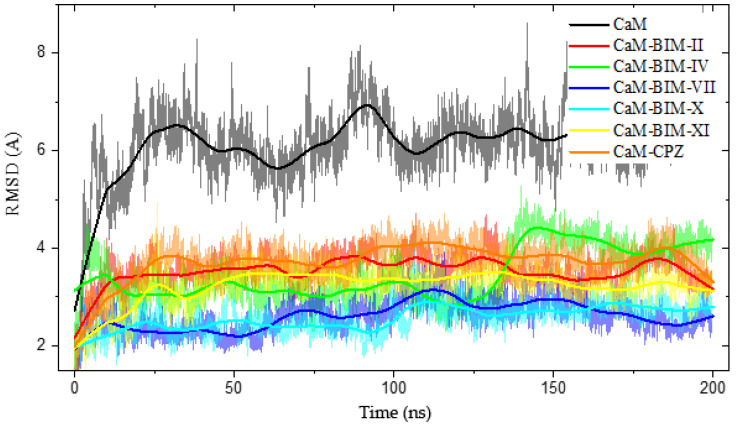
The RMSD & Time plot for 200 ns MD simulation. Shows the differences between closed CaM and CaM-BIM-II, CaM-BIM-IV, CaM-BIM-VII, CaM-BIM-X, CaM-BIM-XI, and CaM-CPZ.

Figure 8, shows the Root Mean Square Deviation (RMSD) as a function of time, where we can observe the difference between CaM without ligand and CaM with BIMs; this difference in RMSD is mainly related to the structural stability of CaM, where all CaM-BIS complexes have a lower RMSD (between 2 and 4 Å), while ligand-free CaM has an RMSD of around 6 Å.

**Figure 9:**
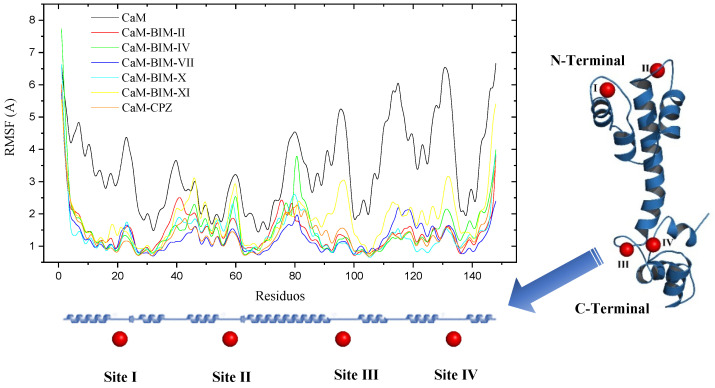
RMSF vs Time of CaM-BIS complexes and three-dimensional model of CaM highlighting calcium ion binding sites in red spheres.

Another parameter we evaluate in MD is the Root Mean Square Fluctuations (RMSF) by amino acids of CaM, where we observe which areas of the protein present greater and lesser flexibility. Figure 9 shows us in a general way that the lobe corresponding to the C-terminal of CaM is considerably more flexible, as well as the zones corresponding to the four calcium binding sites. In the lobe corresponding to the C-Terminal are the amino acids that interact with BIMs in site I, according to docking studies, which makes sense due to the flexibility of this region. Comparing the RMSF of CaM in the absence or presence of the ligands, we can observe that the BIMs and CPZ compounds confer less flexibility to CaM in all its regions, which makes the complexes more stable in general.

The theoretical energy parameters calculated from MD trajectories are shown in Table 3. All the complexes have a negative ΔG, the majority contribution given by the enthalpy component (ΔH). The entropic component (ΔS) is lower in all cases, which is associated with the stability of CaM-Ligand complexes. BIM-VII compound has the lowest ΔG (−49.48 Kcal/mol), which agrees with experimental and docking data where this compound has the best affinity (186.2 and 2.14 nM, respectively). At the other extreme, we have a positive control (CPZ), which has the lowest affinity (492.2 nM) and the highest ΔG of all the ligands studied; this relationship indicates that MD studies are an excellent option to complement our experimental results and to be able to predict good results in subsequent theoretical studies.

### 2.4. Chemoinformatic Analysis

One of the main problems in developing bioactive molecules that could be considered potential drugs is that the final phases of clinical trials may not be satisfactory; one way to reduce this possibility is through chemoinformatic studies since these can help predict the behavior of bioactive molecules in biological systems. Table 4 shows the cheminformatic properties of the BIM compounds, this information based on computational theoretical information predicts some crucial parameters to evaluate the possibility that any of the compounds proposed in this work could be considered a drug candidate. Within these parameters, we can highlight the drug score, which evaluates all the desirable characteristics of a molecule to be considered a drug; the range is from 0 to 1, being better the closer it is to 1; for our compounds, this parameter is found in the range of 0.64 to 0.92. Another parameter to highlight that directly affects a compound’s affinity (power) for possible molecular targets is hydrogen bonds. The BIM series has a greater probability of accepting hydrogen bonds than donating, with BIM-VII being the compound that can accept up to 7, which also agrees with the best affinity and the lowest theoretical binding energy (Docking and MD). Finally, the calculated partition coefficient (cLogP) indicates the degree of hydrophobicity of a molecule; an essential parameter for the absorption of this in biological systems since most drugs have to cross cell membranes to reach molecular targets within cells. The BIMs have a cLogP between 1.55 and 3.02, which are acceptable ranges. In general, this chemoinformatic analysis predicts that the BIM series has a good chance that one of the compounds will be considered a potential anti-CaM drug candidate.

## 3. Materials and Methods

### 3.1. Chemistry

The biosensor (*h*CaM M124C-*mBBr*) was obtained using the methodology described above by González-Andrade, M., and *col*. [21]. Drugs and BIMs were acquired from Sigma (St. Louis, MO, USA) and Santa Cruz Biotechnology, Inc. (Dallas, TX, USA). All other reagents were of analytical grade and purchased from Sigma (St. Louis, MO, USA).

### 3.2. Steady-State Fluorescence

All measurements were conducted with an ISS–PC1 spectrofluorometer (ISS, Champaign, IL, USA) with a sample stirring at 37 °C. *h*CaM M124C-*mBBr* (1 μM) was incubated in buffer (10 mM of potassium acetate [pH 5.1] and 10 μM of CaCl_2_). Fluorescence emission spectra were acquired with excitation and emission slit widths of 4 and 8 nm, respectively. The excitation wavelength was 381 nm, and emission wavelengths of 415 to 550 nm were measured. The fractional degree of saturated *h*CaM M124C-*mBBr* with ligand (*y*) was calculated by changes in fluorescence on ligand binding according to *y* = (F − F_0_)/(F_∞_ − F_0_), where F_∞_ represents the fluorescence intensity at saturation of the ligand, *y* is plotted as a function of the protein/ligand relation (*L*), and apparent dissociation constants (*K*_d_) and stoichiometric (*S*) were obtained by fitting to the equation:

(1) $$y=\frac{\left(1+K_{d}/S+L/S\right)-\sqrt{{\left(1+K_{d}/S+L/S\right)}^{2}-4L/S}}{2}$$

where *y* represents the fractional degree of fluorescence intensity at 470 nm, *K*_d_ is the apparent dissociation constant for the ligands, *L* is the protein/ligand relation, and *S* is stoichiometric. The data were analyzed using the OriginPro version 9.0 64-bit SR2 program (OriginLab, Northampton, MA, USA).

### 3.3. Preparation of Initial Coordinate Files

Coordinates corresponding to the structure of CaM were obtained from the Protein Data Bank (PDB, http://www.rcsb.org (accessed on 1 September 2022)). The CaM-ligands complexes, the X-ray structure of CaM with calcium and TFP named 1LIN.pdb (1LIN, close form of the CaM) refined at 2.0 Å were chosen [34]. Ligands were obtained from the PDB co-crystillized structure, and when the crystals were unavailable, their structures were constructed using HyperChem 8 software. All structures of the ligands were minimized using Gaussian 09, revision A.02 (Gaussian Inc., Wallingford, CT, USA) at the DTF B3LYP/3-21G level of theory. Inhibitors’ partial charges and force field parameters were generated automatically using the *antechamber* program in AmberTools22 [35].

### 3.4. Docking Studies

Docking was conducted using the PDB X-ray structure of the CaM with the ligand TFP (1A29.pdb). We performed a final all-atom refinement of CaM with the idealization application of the Rosetta3.1 release [36]. All compounds were built using the HyperChem 8.0 release program and optimized geometrically using the Gaussian 09 program, revision A.02 (Gaussian Inc., Wallingford, CT, USA) at the DTF B3LYP/3-21G level of theory. Protein and ligands were further prepared using the utilities implemented by AutoDockTools 1.5.4 (https://ccsb.scripps.edu/mgltools/ (accessed on 1 September 2022)). Protein added polar hydrogen atoms, Kollman united-atom partial charges, and to the ligands computing Gasteiger–Marsilli formalism charges, rotatable groups were assigned automatically, as were the active torsions. Blind docking was carried out using AutoDock4 version 4.2 software (http://autodock.scripps.edu/ (accessed on 1 September 2022)) [37,38,39], using default parameters, the Lamarckian genetic algorithm with local search, number of individuals in a population (150), maximum number of energy evaluations (2.5 million), maximum number of generations (27,000), rate of gene mutation (0.02), rate of crossover (0.8) and 100 runs for docking. Electrostatic grid maps were generated for each atom type in the ligands using the auxiliary program AutoGrid4 part of AutoDock4. The initial grid box size was 60 Å × 60 Å × 60 Å in the x, y, and z dimensions. To refine the docking analyses, they were performed in a smaller grid box, with 30 Å × 30 Å × 30 Å dimensions, placed in the ligand. Docking analysis was made with AutoDockTools using cluster analysis and PyMOL [40].

### 3.5. Molecular Dynamics Simulation Studies

Coordinates of the ligands resulting from the docking study were processed with an *antechamber* (a set of auxiliary programs for molecular mechanic studies) to generate suitable topologies for the LEaP module from AmberTools22 [35,41,42,43]. Each structure and complex were subjected to the following protocol: hydrogen and other missing atoms were added using the LEaP module with the parm99 parameter set (PARM99 + frcmod.ff99SB + frcmod.parmbsc0 + OL3 for RNA + ff19SB), Na^+^ counterions were added to neutralize the system, the complexes were then solvated in an octahedral box of explicit TIP3P model water molecules localizing the box limits at 12 Å from the protein surface. MD simulations were performed at 1 atm and 298 K, maintained with the Berendsen barostat and thermostat, using periodic boundary conditions and particle mesh Ewald sums (grid spacing of 1 Å) for treating long-range electrostatic interactions with a 10 Å cutoff for computing direct interactions. SHAKE algorithm was used to satisfy bond constraints, allowing the employment of a 2-fs time step to integrate Newton’s equations as recommended in the Amber package [42,44]. Amber f99SB force field [43,45,46] parameters were used for all residues, and Gaff force field [47,48] parameters were used for the ligands. All calculations were made using graphics processing units (GPU, Tesla V100, NVIDIA Corporation, Santa Clara, CA, USA) accelerated MD engine in AMBER (pmemd.cuda), a program package that runs entirely on CUDA-enabled GPUs [49]. The protocol consisted in performing an optimization of the initial structure, followed by a 50 ps heating step at 298 K, 50 ps for equilibration at constant volume, and 500 ps for equilibration at constant pressure. Several independent 200 ns MD simulations were performed. Frames were saved at 100 ps intervals for subsequent analysis.

### 3.6. Binding Free Energies Calculated by Molecular Mechanics/Poisson Boltzmann Surface Area (MM/PBSA)

This method combines molecular mechanics’ energy with implicit solvation models to calculate binding free energies. In MM/PBSA [50,51], binding free energy (Δ*G*_bind_) between a ligand (L) and a target (T) to form a complex is calculated as:

$$\begin{array}{c} \Delta G_{\mathrm{bind}}=\Delta H-T\Delta S\approx \Delta E_{\mathrm{MM}}+\Delta G_{\mathrm{Sol}}-T\Delta S\\ \Delta E_{\mathrm{MM}}=\Delta E_{\mathrm{Internal}}+\Delta E_{\mathrm{Electrostatic}}+\Delta E_{\mathrm{Vdw}}\\ \Delta G_{\mathrm{Sol}}=\Delta G_{\mathrm{PB}}+\Delta G_{\mathrm{SA}}\; \end{array}$$

where Δ*E*_MM_, Δ*G*_Sol,_ and −*T*Δ*S* are the changes of the gas phase molecular mechanics energy, the solvation free energy, and the conformational entropy upon binding, respectively. Δ*E*_MM_ comprises Δ*E*_Internal_ (bond, angle, and dihedral energies), Δ*E*_Electrostatic_ (electrostatic energies), and Δ*E*_Vdw_ (van der Waals energies). Δ*G*_Solv_ is the sum of electrostatic solvation energy (polar contribution) (Δ*G*_PB_) and non-electrostatic solvation component (non-polar contribution) (Δ*G*_SA_). Polar contribution is calculated using the Poisson-Boltzmann surface area model, while non-polar energy is estimated from the solvent-accessible surface area (*SASA*). Conformational entropy change (−*T*Δ*S*) was computed by normal mode analysis from a set of conformational snapshots taken from the MD simulations [50,52,53].

### 3.7. Trajectory Analysis

Analyses were done using CPPTRAJ [49] part of AmberTools22 utilities and Origin 9.0. First, the RMSD and Root Mean Square Fluctuations (RMSF) calculations were made, considering the C, CA, and N; for the distances, only CA was used. Charts were built with Origin 9.0, and trends were adjusted with smooth function processing (method Lowess).

All calculations were made using a system HP Cluster Platform 3000SL, supercomputer “MIZTLI” with a processing capacity of 118 TFlop/s. It has 5312 Intel E5-2670 processing cores, 16 NVIDIA m2090 cards, GPU Tesla V100, a total RAM of 15,000 Gbytes, and a mass storage system of 750 Terabytes (http://www.super.unam.mx/ (accessed on 1 September 2022)).

## 4. Conclusions

In this work, a series of compounds called BIM were evaluated as possible inhibitors of CaM protein, which have not been described in the literature for this purpose. Binding assays using the direct *h*CaM-M124C-*mBBr* biosensor indicate that BIMs bind to CaM protein with a *K*_d_ in the nM order of magnitud, better than most classical inhibitors. Complementary pharmacological or physiological studies can be carried out in the future, depending on the metabolic pathway associated with the CaM that is to be studied. Furthermore, docking results complement and confirm experimental results detailing the interactions at a molecular level of the BIMs with CaM. At the same time, MD describes the structural stability and theoretical thermodynamic parameters associated with the Ca^2+^-CaM-BIM complexes. Finally, chemoinformatic analyzes predict some favorable data to consider this series of BIMs compounds as possible CaM inhibitors to be considered as anti-CaM drugs.

## Figures and Tables

**Table 1:** Experimental and theoretical binding properties of Ca^2+^-CaM-BIMs complexes.

	Experimental Studies		Docking Studies			
	*K*_d_ (nM)	Stoichiometry	*K*_i_ (nM)			
			Site I	Site II	Site III	Site IV
Ca^2+^-CaM -BIM-II	257.8 ± 5.5	2.5 ± 0.2	109.79	161.58	-	-
Ca^2+^-CaM -BIM-IV	223.8 ± 3.7	3.7 ± 0.2	17.10	3390	7450	-
Ca^2+^-CaM -BIM-VII	186.2 ± 4.1	4.4 ± 0.1	2.14	26.95	52.43	263.87
Ca^2+^-CaM -BIM-X	205.2 ± 3.8	4.3 ± 0.1	20.36	59.39	71.65	217.1
Ca^2+^-CaM -BIM-XI	239.0 ± 5.0	3.4 ± 0.1	9.66	55.96	75.12	191.71
Ca^2+^-CaM -CPZ	492.2 ± 4.6	3.4 ± 0.1	715.65	1169	1640	-
Ca^2+^-CaM -TFP	532.7 ± 74.2 ^1^	1.6 ± 0.2	384	707	959	976

^1^ This data was taken from previous experiments of the same working group [33].

**Table 2:** Interactions of BIM compounds with CaM from the site I docking.

	Interaction Residuals
Ca^2+^-CaM-BIM-II	Leu39, Ala88, Val91, Phe141, Phe92, Ile100, Leu105, Val108, Met109, Leu112, Met124, Ile125, Glu127, Val136
Ca^2+^-CaM-BIM-IV	Glu127, Ile125, Met124, Leu105, Val108, Met109, Leu112, Leu39, Phe141, Phe92, Ile100, Val136
Ca^2+^-CaM-BIM-VII	Glu7, Glu11, Glu14, Phe92, Phe141, Phe144, Leu105, Ile100, Val136, Met124, Ile125, Met109, Glu127
Ca^2+^-CaM-BIM-X	Met124, Glu127, Phe92, Ile100, Leu112, Met109, Val108, Leu39, Phe141, Met144, Met145
Ca^2+^-CaM-BIM-XI	Val136, Phe92, Ile100, Ala128, Glu127, Ile125, Met124, Lys148, Ala147, Met145, Met144
Ca^2+^-CaM -CPZ	Glu127, Ile125, Met124, Leu105, Val136, Ile100, Phe92, Phe141, Met144

**Table 3:** Theoretical energy parameters of the Ca^2+^-CaM-BIM complexes from the MD studies.

	ΔG (Kcal/mol)	ΔH (Kcal/mol)	ΔS (Kcal/mol)
Ca^2+^-CaM-BIM-II	−42.73 ± 9.64	−67.61 ± 3.88	−24.87 ± 8.82
Ca^2+^-CaM-BIM-IV	−30.25 ± 3.91	−47.61 ± 3.29	−17.36 ± 2.10
Ca^2+^-CaM-BIM-VII	−49.48 ± 6.92	−72.65 ± 5.11	−23.17 ± 4.67
Ca^2+^-CaM-BIM-X	−23.57 ± 5.19	−49.64 ± 3.31	−26.06 ± 4.00
Ca^2+^-CaM-BIM-XI	−45.47 ± 9.67	−71.21 ± 5.23	−25.74 ± 8.13
Ca^2+^-CaM-CPZ	−16.77 ± 5.77	−35.01 ± 4.53	−18.24 ± 3.58

**Table 4:** Chemoinformatic properties of the BIM and CPZ compounds.

Compound	BIM-II	BIM-IV	BIM-VII	BIM-X	BIM-XI	CPZ
cLogP	2.43	1.58	1.55	2.4	3.02	4.61
Solubility (LogS)	−3.38	−3.55	−2.55	−3.23	−2.78	−4.8
Molecular weight	438.53	327.34	453.54	425.5	452.56	318.87
Druglikeness	7.73	4.21	6.81	6.65	8.05	8.38
H bond acceptor	6	5	7	6	6	2
H bond donor	2	3	3	2	1	0
Stereocenters	1	0	0	1	1	0
Rotatable bonds	5	2	6	3	4	4
Drug score	0.81	0.92	0.64	0.83	0.77	0.45

Data were calculated using the OSIRIS server http://www.cheminfo.org/Chemistry/Cheminformatics/Property_explorer/index.html (accessed on 1 September 2022).

## Data Availability

Not applicable.
